# CA-VLN: Collaborative Agents in MLLM-Powered Visual-Language Navigation

**DOI:** 10.3390/s26041254

**Published:** 2026-02-14

**Authors:** Ruolin Zhu, Shaobin Li, Zixing Zhu, Jing Jia, Min Yang

**Affiliations:** 1 School of Information and Communication Engineering, Communication University of China, Beijing 100024, China; 202210081000067@cuc.edu.cn (R.Z.); 202220081001036@cuc.edu.cn (Z.Z.); jingj78@mails.cuc.edu.cn (J.J.); 2 School of Artificial Intelligence, Beijing University of Posts and Telecommunications, Beijing 100088, China; 2016110924@bupt.cn

**Keywords:** multimodal feature fusion, world knowledge, episodic memory, hierarchical history

## Abstract

Generalization to unseen environments remains a fundamental challenge in Vision-Language Navigation. To tackle this issue, we propose a novel framework that leverages world knowledge embedded within Multimodal Large Language Models. We introduce Collaborative Agents in Visual-Language Navigation (CA-VLN), a framework based on a dual-agent architecture. This architecture comprises a Knowledge Agent, which infuses the action prediction process with semantic context and commonsense reasoning, and a Hierarchical History Agent, which constructs a detailed episodic memory to enable long-horizon planning. The collaboration between these agents facilitates a dynamic interplay between high-level semantic understanding and grounded episodic experience. Extensive experiments on the R2R, REVERIE and SOON datasets demonstrate that our model achieves state-of-the-art performance, significantly improving generalization and navigation success in previously unobserved environments.

## 1. Introduction

Visual-Language Navigation (VLN), a crucial task in embodied intelligence, challenges an agent to navigate complex environments by interpreting natural language instructions. The ability to ground high-level commands in visual observations is crucial for real-world applications, from domestic robotics to autonomous exploration. Despite significant progress, a fundamental obstacle continues to limit the practical deployment of VLN agents: generalizing navigation strategies to novel, unseen environments. This challenge stems from the difficulty of transferring learned behaviors to unfamiliar layouts, objects, and visual appearances, a core problem that our work aims to address.

Previous methods have consistently suffered from these shortcomings: data scarcity, shallow integration of multimodal features, and lack of historical context, which are vital for long-horizon navigation tasks. The recent emergence of Multimodal Large Language Models (MLLMs), known for their vast world knowledge and sophisticated reasoning abilities, offers a promising approach to solve this generalization problem. In theory, MLLMs can provide the common sense understanding required to interpret ambiguous instructions and reason about unseen scenes, which has driven their adoption in embodied intelligence tasks [1,2,3]. Some studies have also explored integrating MLLMs to convert visual features into textual representations, thus facilitating action decisions [4,5,6,7]. However, a fundamental domain gap persists between MLLMs and VLN, rendering direct application of MLLMs for action decisions. 3D modeling of real-world navigation scenarios also faces technical challenges: the limited fidelity of simulated environments constrains agents’ generalization capabilities in unfamiliar real-world settings, while the construction of instruction-trajectory datasets remains labor-intensive and costly. Most existing VLN tasks [8,9,10] focus on fine-grained instructions and prioritize the navigation process itself to facilitate agents’ reasoning training. However, real-world users typically issue coarse-grained, goal-oriented instructions (e.g., “Go to the kitchen faucet and fill a glass with water”), such instructions require agents to explore independently and demanding stronger visual understanding and path-planning capabilities. To address this discrepancy, this paper proposes a text-instruction-driven stepwise reasoning approach. Fine-grained instructions support single-step action prediction, while coarse-grained, goal-oriented, or ambiguous instructions are appropriately extended using visual information from observation points.

We introduce the Collaborative Agents for Visual-Language Navigation, a novel framework that implements a dual-agent architecture. Our approach synergizes the strengths of MLLMs with the demands of embodied navigation by decomposing the task into two specialized collaborative roles: one is a Knowledge Agent, which leverages an MLLM for high-level semantic reasoning. It infuses the decision-making process with rich contextual understanding, interpreting the environment and instructions through a lens of world knowledge. The other is a History Agent, which maintains a detailed episodic memory. This agent grounds the navigation process by tracking visited locations, recording observations, and managing the agent’s trajectory, enabling informed long-term planning and backtracking. The core innovation of CA-VLN lies in the dynamic interplay between these two agents, facilitating a powerful combination of high-level semantic abstraction and grounded in situ experience. This synergy allows the agent to navigate robustly, especially in unfamiliar settings. Our main contributions can be summarized as follows.

- **Collaborative Agents**: Knowledge Reasoning Agent is a knowledge-enhanced instruction framework. The integration of world-knowledge and landmark information into fine-grained instructions facilitates agents to follow instructions better. Hierarchical History Agent is an episodic memory enhancement module that equips agents with long-term memory through episodic memory retrieval, enabling navigation backtracking of visited viewpoints. Through collaboration, these two agents facilitate a dynamic interplay between high-level semantic cognition and grounded episodic memory.
- **Multimodal Fusion Module**: We propose a Knowledge-Guided Multimodal Fusion Module that leverages knowledge and instructions to guide visual feature extraction while dynamically reweighting the information content across diverse viewpoints.
- **Comprehensive Experimental Validation**: We conducted extensive experiments and achieve the sota performance on multiple benchmark datasets. The results demonstrate the robustness and adaptability of our model, confirming its effectiveness in navigation tasks.

## 2. Related Work

### 2.1. VLN

Generalization capability remains a central challenge in the VLN domain. Effective data augmentation strategies [11,12,13] and the incorporation of high-fidelity data scenarios [14,15,16] have mitigated the problem of data scarcity. While some studies focus on expanding instructional datasets [17], others optimize pre-training tasks by introducing detour path configurations [18]. Inspired by advances in multimodal representation learning, numerous enhancements to VLN pre-training frameworks [19,20,21] have been proposed to strengthen models’ ability to encode multimodal features.

Recent advances in graph-based Transformer architectures demonstrate remarkable efficacy in discrete-scene navigation, as exemplified by topological graph representations [22,23,24] and bird’s-eye view modeling [25]. Dual-scale Graph Transformer (DUET) [22] has explicitly shown that graph-based Transformers embody critical competencies for path planning and multimodal feature fusion, leveraging navigational memory to enable long-horizon action prediction. MapGPT [26] introduces a graph-guided topological adaptive planning mechanism, which activates the pathfinding capabilities of the GPT models through structured spatial guidance. Path planning methodologies have been shown to empower agents with enhanced long-term action prediction and navigation state transition modeling [27,28,29]. Anderson et al. Adaptive Cross-Modal Experts Network (ACME) [30] introduces a dynamic expert model, adaptively selects the most suitable processing expert. Then, it employs an uncertainty-driven fusion module that calculates the confidence of both coarse-grained and fine-grained information, dynamically adjusting their weights in the final decision.

### 2.2. VLN with LLM

To tackle the zero-shot challenge in unseen environments, recent investigations have integrated MLLMs to augment agents’ multimodal comprehension and inferential capabilities. GPT-based architectures have been deployed to provide auxiliary navigation guidance to downstream navigation agents [31,32]. Using the zero-shot proficiency of MLLMs [33], some studies directly employ MLLMs as a navigation agent, where prompt-generated output dictates action decisions in navigation tasks [4,5,34]. Alternatively, zero-shot navigation has been realized through multi-expert deliberation frameworks that optimize decision processes [35]. However, these algorithms exhibit significant performance gaps compared to supervised VLN approaches, primarily attributed to the substantial domain disparity between large model training corpora and VLN-specific datasets.

Consequently, emerging research has focused on domain-specific fine-tuning of MLLMs to enable their role as world models and navigation reasoning agents. NaviLLM [36] seeks to establish a general model for embodied navigation by fine-tuning Vicuna-7B model [37] using multitask datasets from the embodied domain. EmbodiedGPT [38] has constructed a large-scale embodied planning corpus, employing prefix tuning to adapt 7B-class large models to navigation tasks. NavCoT [6] introduces a parameter-efficient training paradigm, fine-tuning two open-source LLaMA-Adapter [39] and LLaMA2 [40] to facilitate the integration of LLM with VLN tasks at reduced computational costs.

### 2.3. VLN with Knowledge

To enhance the interpretability of action prediction in existing VLN algorithms, recent studies have proposed integrating common sense knowledge to facilitate action prediction. Early works leveraged graph neural networks to model structured object relationships in scenes, reinforcing cross-modal interactions between visuals and language [22,41,42], or constructed offline unstructured knowledge bases for contextual knowledge retrieval [43,44]. Recent advancements like Esc [45] employ Grounded Language-image Pre-training (GLIP) for object detection and description, leveraging large language models (LLMs) to generate inter-entity relationships for zero-shot navigation. Demand-driven Navigation (DDN) [46] extracts commonsense knowledge from MLLMs to derive the textual attribute features of objects. State-of-the-art approaches convert visual observations into natural language descriptions [4,5] and fine-tune LLaMA-7B to select actions based on instructions and scene narratives [47]. KERM [48] constructs a knowledge base from annotations of the Visual Genome [49] Dataset. However, its reliance on pre-constructed knowledge bases can still be a limitation when encountering completely novel out-of-domain scenarios. Our work, while also leveraging external knowledge, focuses on generating this knowledge on-the-fly using MLLMs, thus reducing dependency on static knowledge bases and combining it with a hierarchical memory structure for long-horizon tasks.

## 3. Method

### 3.1. Overall Architecture

**Figure 1:**
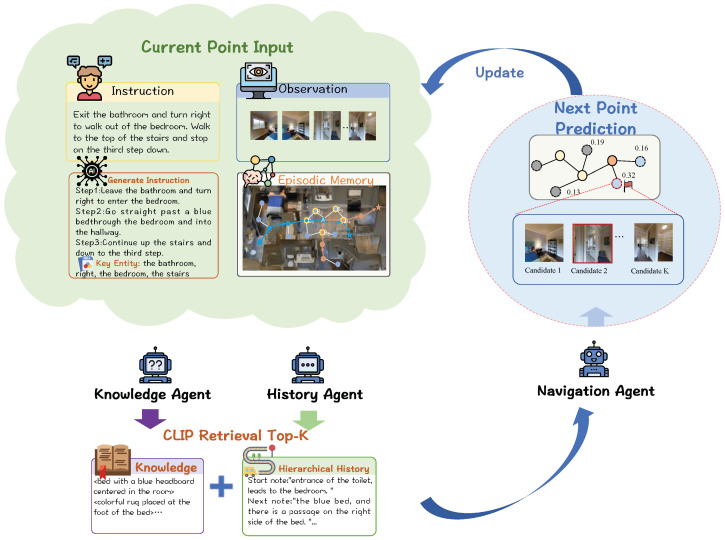
Overall block diagram of the collaboration of multiple Agents for visual-language navigation, one is knowledge Agent and Hierarchical History Agent. And a multimodal feature retrieval method is adopted to screen out the Top-K relevant text for action prediction.

This paper exploits the knowledge and episodic memory of MLLMs to enhance a multimodal navigation framework, as depicted in Figure 1.

**Figure 2:**
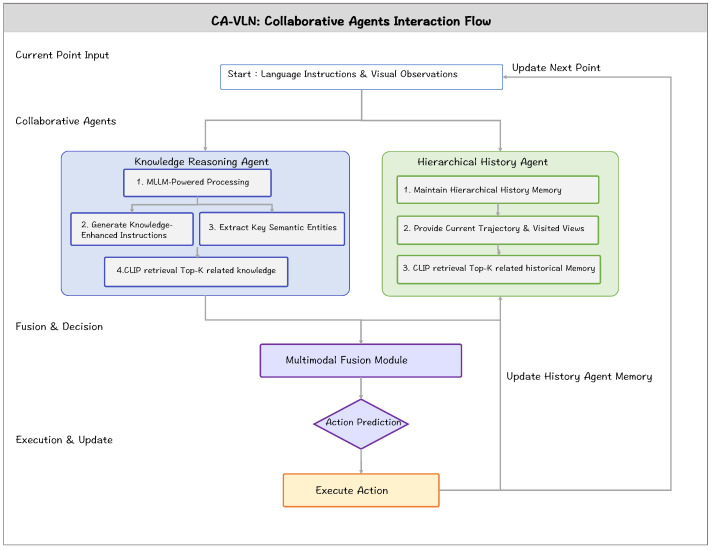
Information flow and interaction process between the Knowledge Agent and the Hierarchical History Agent.

The collaboration process between Knowledge Agent and Hierarchical History Agent starts with the Knowledge Agent driven by MLLMs, which is responsible for parsing instructions and environmental observations to generate knowledge-enhanced guidance information. Subsequently, this guidance information, along with the agent’s previous state, is processed by the Hierarchical History Agent. This agent makes the final action decision by maintaining episodic memory and semantic memory, as shown in Figure 2.

### 3.2. Knowledge Reasoning Agent

The Knowledge Reasoning Agent guides the extraction of relevant visual features. It directs the agent’s focus toward significant landmarks and integrates semantic context from world knowledge, helping the agent make more informed decisions.

#### 3.2.1. Knowledge-Enhanced Instruction

To enhance comprehension of dense instructions, we generate stepwise instructions for decomposing navigation tasks. Departing from traditional shortest-path planning approaches that rely exclusively on start-end viewpoints, the agent predicts N-step sequential actions to reach the target. To optimize visual-instruction alignment, we convert visual views into the corresponding language instruction descriptions.

**Figure 3:**
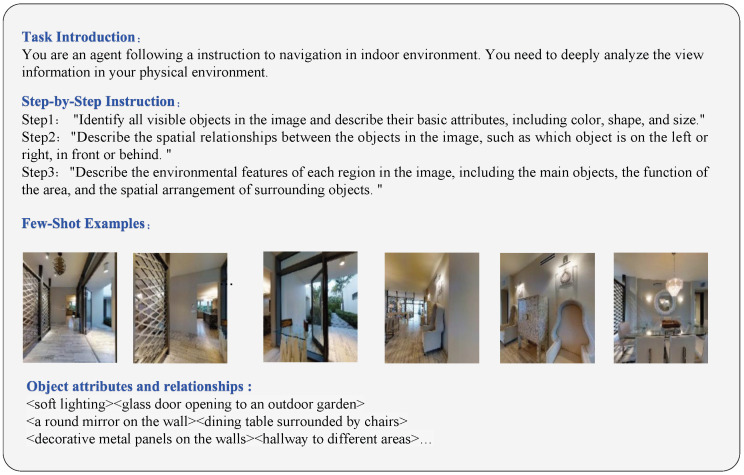
Step-by-step prompt words to generate new navigation instructions and key entities in the instructions.

We adopt different strategies for knowledge generation in the training and inference phases. During training, we feed the original instructions and the annotated ground-truth trajectory into the Multimodal Large Language Model (MLLM). This generates precise stepwise instructions and extracts key semantic entities to serve as supplementary supervision. During inference, however, ground-truth data is unavailable. Therefore, the agent generates knowledge using only the original instruction, real-time visual observations, and the trajectory history of the current step. A critical aspect of constructing instructions involves the deliberate design of effective prompting strategies, which guide MLLMs to generate semantically coherent instructions. The process is illustrated in Figure 3.

The object attributes and spatial relationships generated by MLLMs constitute the essential knowledge we focus on. During the navigation process, knowledge is updated to reflect the dynamic environment. We use the CLIP text encoder to embed the current instruction and retrieve the Top-K most semantically related facts (e.g., “Dining table is near the kitchen”) via cosine similarity to augment the visual features. During inference, given a query q∈Q, the agent performs a retrieval operation Ψ to access the relevant knowledge sequence. This process is formulated as:

(1) $$K_{t}{|}_{q}=\Psi \left(q,K_{t}\right)$$

where Ψ denotes the retrieval strategy [50], and $K_{t}{|}_{q}\subseteq K_{t}$ represents the retrieved subset of the knowledge pool of current task. The query is the instruction of the current task.

#### 3.2.2. Entity-Guided Feature Enhancement

Position embedding $E_{i}^{P}\in R^{d_{m}}$ is employed to encode sequential relationships. For text, we adopt Bidirectional Encoder Representations from Transformers (BERT) [51] to extract text features. The position embedding is concatenated with entity embeddings, which can help the model to capture spatial relationships between entities. This concatenation mechanism is formulated as follows.

(2) $$\varepsilon_{c}^{W}=\mathrm{BERT}\left(w_{\mathrm{merge}},E_{i}^{P},E_{o}^{T}\right)$$

To enhance the representation of the key region within instructions based on their semantic correlation, multi-head attention is used to update the contextual features, emphasizing the semantic representation of the critical region.

### 3.3. Multimodal Fusion and Action Prediction

**Figure 4:**
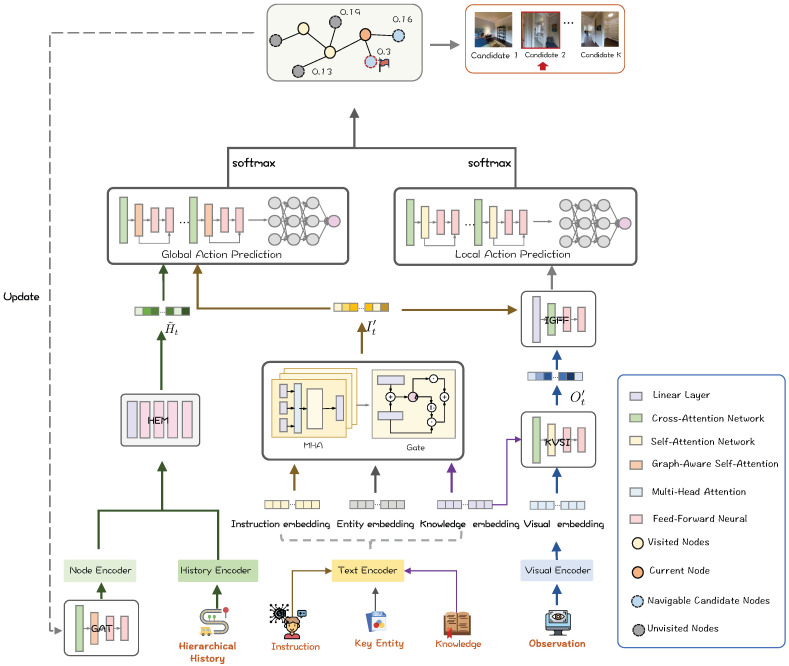
The architecture presents the multimodal fusion approaches for input features. These features are respectively utilized in global or local prediction according to the distinct characteristics of each modal feature.

After acquiring multimodal features, Multimodal Fusion Module is also the core components of the architecture, as depicted in Figure 4. Knowledge and images contain a large amount of detailed information, which is conducive to making fine-grained local prediction. Meanwhile, hierarchical history is more suitable for conducting coarse-grained prediction, as a summary of global information. To better clarify the fusion process, we present the pseudocode of our multi-modal fusion algorithm, as illustrated in Algorithm A1 in Appendix A.

#### 3.3.1. Instruction-Guided Feature Fusion

The Instruction-Guided Feature Fusion (IGFF) Module leverages instructions to guide visual feature extraction, directing agents to focus on semantically relevant landmarks within the visual scene. We extract the global semantic feature ${\hat{W}}_{0}$ by projecting the [CLS] token from the instruction embeddings *W* via a linear layer defined as ${\hat{W}}_{0}=W_{L}\cdot W_{cls}$. To identify relevant landmarks, we compute attention weights $\eta_{i}$ that measure the responsiveness of each visual feature $o_{i}$ to the global instruction context:

(3) $$\eta_{i}=\mathrm{softmax}\left(\frac{o_{i}W_{q}{\hat{W}}_{0}^{T}}{\sqrt{d}}\right),\;{\bar{o}}_{i}=\eta_{i}o_{i}$$

where ${\bar{o}}_{i}$ denotes the instruction-guided visual representation. This ensures the agent focuses on landmarks mentioned in the text (e.g., “kitchen”, “stairs”).

#### 3.3.2. Knowledge-Aware Visual Semantic Interactor

We propose the Knowledge-aware Visual Semantic Interactor Module (KVSI), which fuses visual features with semantic knowledge to enhance the scene understanding capabilities of the agent. A cross-modal attention mechanism fuses these modalities, where attention scores $a_{ij}$ are calculated between visual queries and knowledge keys. The knowledge-enhanced features $O_{t}^{\prime }$ are obtained via a weighted sum:

(4) $$a_{ij}=\mathrm{softmax}\left(\frac{QK^{T}}{\sqrt{d}}\right),\;o_{i}^{\prime }=\sum_{j}a_{ij}\cdot V_{j}$$

To capture spatial relationships, $o_{i}^{\prime }$ undergoes a self-attention refinement process yielding $O_{t}^{\prime }=\sum_{j}\beta_{ij}V_{j}$, where $\beta_{ij}$ represents the self-attention weights. Finally, to address information imbalance across viewpoints, we employ an Adaptive View Weighting mechanism. We concatenate the refined visual feature $O_{t}^{\prime }$ with knowledge features $K_{t}^{\prime }$ to compute an importance score $s_{i}$ relative to the history context. The final contextually refined representation $V_{context}$ is aggregated using normalized weights $w_{i}$:

(5) $$w_{i}=\frac{e^{s_{i}}}{\sum_{j}e^{s_{j}}},\;V_{context}=\sum_{i}w_{i}O_{t,i}^{\prime \prime }$$

This hierarchical fusion ensures the agent focuses on the most salient visual-semantic cues for navigation.

#### 3.3.3. Adaptive Weighting and Action Selection

The final action decision requires balancing the immediate visual-semantic feature $O_{t}^{\prime }$ with the long-term trajectory context ${\tilde{H}}_{t}$. We introduce an Adaptive View Weighting Mechanism to compute a navigation score $S_{i}$ for each candidate viewpoint.The fusion process follows a three-step operation:

1. **Feature Concatenation:** For each candidate *i*, we construct a unified decision vector $F_{i}$ by concatenating the instruction-guided visual feature, the knowledge feature, and the global history context:
  (6) $$F_{i}=\left[O_{t,i}^{\prime };{\tilde{H}}_{t}\right]$$
2. **Attention Score Computation:** We utilize a learnable scoring function (implemented as a two-layer MLP) to project the unified vector into a scalar importance score:
  (7) $$s_{i}=\mathrm{MLP}\left(F_{i}\right)$$
3. **Action Prediction:** The raw scores are normalized via a Softmax function to produce the probability distribution $P_{t}$ over all candidate actions:
  (8) $$P\left(a_{t}=i|V_{t},W,{\tilde{H}}_{t}\right)=\frac{\exp(s_{i})}{\sum_{j=1}^{K}\exp\left(s_{j}\right)}$$

### 3.4. Hierarchical History Agent

#### 3.4.1. Hierarchical Historical Construction and Retrieval

The Hierarchical Historical Retrieval (HHR) transforms historical navigation sequences into hierarchical descriptions that encapsulate comprehensive environmental knowledge. This method is divided into two distinct levels. The first level constitutes a viewpoint hierarchy, where LLaVA generates a scene description for each viewpoint. To prevent memory explosion during hovering or repetitive movements, the historical memory is built incrementally rather than storing every individual view. A new node is added to the memory graph only when the agent advances to a new observation point. This ensures an efficient and accurate spatial representation. The independent viewpoint descriptions $D_{vp}$ are formulated as follows.

(9) $$D_{vp}=\left\{T_{vp},F_{vp},C_{vp}\right\}$$

**Figure 5:**
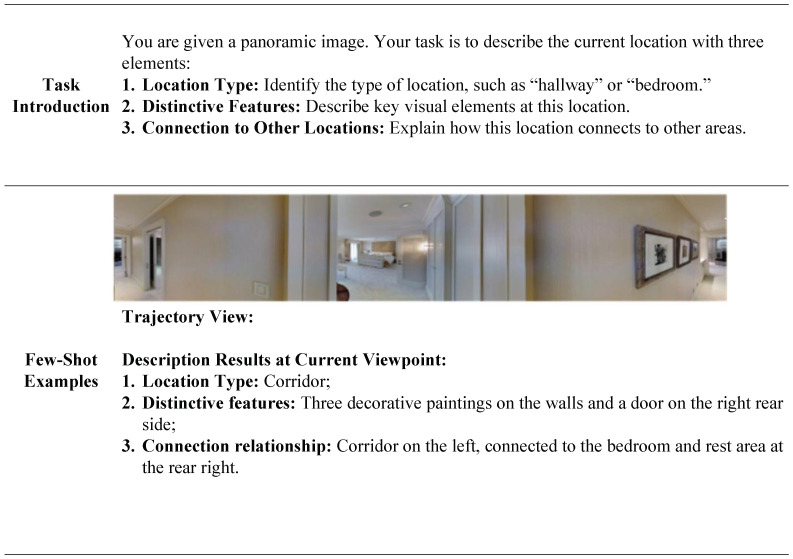
Example of historical memory unit generation.

$T_{vp}$ represents the location type (e.g., “hallway” or “bedroom”). $F_{vp}$ denotes the distinctive feature(key visual captions), and $C_{vp}$ represents the connection relationship to other areas. To clarify the meaning of these elements, we provide an example in Figure 5.

The second level constitutes a path hierarchy that concatenates viewpoint descriptions along the temporal dimension. The path hierarchy preserves the spatiotemporal continuity of historical navigation trajectories while injecting semantic information such as scene attributes and spatial relationships. The complete historical path description S(P) of the current viewpoint is defined.

(10) $$S\left(P\right)=f\left(D_{v_{1}},D_{v_{2}},\ldots ,D_{v_{n-1}}\right),$$

(11) $$f=\left\{\begin{array}{cc} \mathrm{None}\;! & t=1\\ \mathrm{The}\;\mathrm{starting}\;\mathrm{point}\;\mathrm{is}∶\;D_{vp_{1}}!\mathrm{passing}∶\;\sum_{i=2}^{t-1}D_{vp_{i}}, & t>1 \end{array}\right.$$

*f* is a concatenation function that concatenates the descriptions of each viewpoint. When t=1, the historical path remains empty; when t>1, the template sequentially concatenates viewpoint descriptions. To achieve effective fusion of semantics and distinctive features, MLLMs are employed for generating semantically enhanced historical representations. This method effectively enhances the semantic context of historical representations and addresses the contextual association deficiencies in graph node representations.

To further model the agent’s reasoning, the entire retrieval process is represented as a conditional generation function *F*. As shown in the following equation, the output *y* is generated based on both the query and the retrieved memory.

(12) $$y=F(q,H_{t}{|}_{q})$$

where $H_{t}{|}_{q}\subseteq H_{t}$ represents the retrieved subset of the history memory and retrieval strategy [50] is the same as that for knowledge retrieval. This approach allows the agent to dynamically focus on task-relevant history, thereby improving decision-making accuracy in complex environments.

#### 3.4.2. History Enhancement Module

This section proposes History Enhancement Module (HEM) via episodic memory to balance the accuracy and efficiency of navigation. Episodic memory provides long-term memory capabilities, enabling agents to recall visited viewpoints. When an agent enters a new scene *Y*, both the memory nodes $V_{Y}^{\left(0\right)}$ and the edges $E_{Y}^{\left(0\right)}$ are initially empty. At each step, the episodic graph is updated with the visited nodes and edges. Node embeddings are incorporated into the episodic graph, where each node feature represents the average of the visual features of adjacent nodes $V_{i}$. After constructing the episodic memory topological graph, episodic memory can be used to enhance the historical representation. The agent retrieves relevant memory $m_{s}$ associated with the current node and adjacent nodes from episodic memory $G^{-1}$. The node feature representation is enhanced with memory representations as follows. A Multi-Layer Perceptron (MLP) is employed to perform non-linear fusion of these features.

(13) $${\tilde{V}}_{t}=\mathrm{MLP}\left(\left[V_{t};m_{s}\right]\right);{\tilde{v}}_{i}=\mathrm{MLP}\left(\left[v_{i};m_{s}\right]\right)$$

(14) $$\tilde{v}=W_{2}\mathrm{ReLU}\left(W_{1}x+b_{1}\right)+b_{2}$$

Due to the complex relationships among the nodes, Graph-Aware Transformer (GAT) is used to establish a global dependency relationship and expand the perception domain. Each GAT layer includes a cross-attention layer, which is used to model the relationship between nodes, and a self-attention layer for encoding the topological layout.

(15) $${\tilde{h}}_{t}^{\prime }=\mathrm{GAT}\left({\tilde{h}}_{t}\right)=\mathrm{softmax}\left(\frac{\left({\tilde{h}}_{t}W_{q}\right){\left({\tilde{h}}_{t}W_{k}\right)}^{T}}{\sqrt{d}}+M\right){\tilde{h}}_{t}W_{v}$$

(16) $$M=DW_{a}+b_{d}$$

Ultimately, the historical information ${\tilde{H}}_{t}$ at the current step is the set of nodes enhanced by episodic memory ${\tilde{H}}_{t}=\left\{{\tilde{h}}_{1}^{\prime },{\tilde{h}}_{2}^{\prime },\ldots ,{\tilde{h}}_{t-1}^{\prime }\right\}$.

## 4. Experiments

### 4.1. Datasets

Three public datasets, such as REVERIE, R2R, and SOON, are selected for evaluation. R2R [8] contains 7189 instructions, with an average length of 32 words. R2R is built on 90 real-world indoor scenes, and the agent only requires predicting the correct view. REVERIE [52] provides high-level instructions, which have an average length of 21 words. Predefined object bounding boxes are provided; the agent is tasked with selecting the correct bounding box from the candidate options. Built on the Foundation for Anywhere-to-Object (FAO), SOON [53] draws on 3848 instructions. The key challenge is the provision of more long-term trajectories, most consisting of 8 steps or fewer. Three datasets differ in task and thus vary in the difficulty level of navigation.

### 4.2. Evaluation Metrics

We use the standard R2R and REVERIE task performance metrics. Navigation Error (NE), the average distance between the agent’s final position and the goal in meters; Success Rate (SR), the ratio of trajectories in which the agent stopped within 3 m of the goal; Oracle Success Rate (OSR), the ratio of trajectories in which the agent stopped within 3 m of the goal with a view of the goal; and success weighted by the normalized inverse of the path length (SPL); Relative Grounding Success Rate (RGSR) and Relative Grounding Success weighted by Path Length (RGSPL) are two important metrics used to evaluate a model to achieve semantic alignment between instructions and objects in the environment.

### 4.3. Implementation Details and Experimental Setup

**Hyperparameters.** All experiments are run on an NVIDIA Tesla A40 (48 GB VRAM). The constructed model interacts with the Matterport3D simulation environment. Both the visual feature dimension and the text feature dimension are set to 768 d, and the hidden layer size is 768 d. The AdamW optimizer is used, and the learning rate is set to 5 × 10^−5^. The dropout ratio is 0.5. Top-K is set to 5 for the R2R and SOON datasets and 3 for the REVERIE dataset. The best model is selected by SR on val unseen split. Other hyperparameters are set the same as in DUET [22].

**Architectural Details.** We use CLIP-ViT-B/16 [54] to extract visual features for both VLN training and retrieval tasks. For object detector, we use the same as in DUET [22]. The core of our architecture consists of the Knowledge Reasoning Agent and the Hierarchical History Agent, which are based on LLaVA2-7B [55]. The input to the Multimodal Fusion Module has a dimension of 1536, resulting from the concatenation of the 768 d visual feature and the 768 d knowledge feature.

**Model Parameters.** The total number of parameters in our CA-VLN model is 7B. First, we train the Knowledge Agent and Hierarchical History Agent using the Low-Rank Adaptation (LoRA) [56], where the number of trainable parameters is constrained within 10 million. Subsequently, we conduct the end-to-end training, integrating the fusion module and prediction module into the joint training framework while the agents are frozen, where the number of trainable parameters is constrained within 10 million. Initialization of fusion module and prediction module is performed with the pretrained LXMERT [57].

### 4.4. Comparison to State-of-the-Art Methods

As detailed in Table 1, CA-VLN delivers substantial performance improvements in the R2R validation set. For unseen scenes, CA-VLN achieves a 73.31% SR (+1.79%) and 61.95% SPL (+1.53%), demonstrating how the synergistic integration of knowledge and episodic memory enhancement boosts agent understanding of complex scenes. CA-VLN exhibits a 1.02% gain in SR and a 1.63% gain in SPL on the test set. Despite marginal improvements, CA-VLN demonstrates enhanced task efficacy, validating its robust generalization and suitability for unseen scenes.

Table 2 is experiments on the REVERIE dataset. On the unseen validation set, SR reaches 50.99%. For the object localization task, RGSR is 35.34%, an increase of 2.85%. On the test set, SR is 53.43% and OSR is 57.61%, with increases of 1.01% and 0.74%, respectively. Our model can effectively capture key entities correlation, thereby achieving the cooperative optimization of path planning and target localization in complex navigation tasks. The global path information provided by HEM module may guide the agent to adjust the path multiple times, and the episodic memory enhances the global exploration ability and expands the exploration range, which may lead to an increase in the length of the navigation path.

To verify the generalization ability, our model is experimented with the SOON dataset with higher spatial complexity, as shown in Table 3. The results show that in the unseen validation set, the SR reaches 37.32%, which is 1.02% higher than that of the baseline. In the unseen test set, the SR reaches 34.35% and the SPL reaches 22.64%, with an increase of 1.12% and 1.49%. For the SOON dataset, which has higher requirements for language understanding and target localization capabilities, CA-VLN also has significant improvements.

### 4.5. Ablation Studies

After extracting knowledge and entities, visual semantics is further enhanced, incorporating KVSI and IGFF to fully integrate factual knowledge, views, and instructions. Ablation experiments are conducted on the REVERIE to validate the efficacy of each module, as detailed in Table 4 and Table 5.

Experimental results demonstrate that KVSI improves the SR by 3.3%, providing empirical evidence of its efficacy in modeling latent relationships between visual features and objects. Knowledge information supplements the high-level semantic details absent from visual representations, thereby bridging the semantic gap in visual understanding. IGFF yields an improvement of 3.88% in SR over the baseline, highlighting its role in aligning visual features with instructions through contextual conditioning. Synergistic effects between KVSI and IGFF lead to substantial performance gains for the navigation model.

When all two modules are integrated, the model achieves a 4.53% improvement in SR, a 2.36% enhancement in SPL, and a 2.88% boost in RGSR, outperforming the cumulative effects of individual modules. This indicates that cross-module information exchange facilitates superior alignment between visual features and instructions. The refined visual representation quality further reinforces navigation success, demonstrating the complementary advantages of multimodal feature fusion.

As shown in the results of Table 6, introducing HHR enriches the ability to express historical information. SR increases by 0.51%, and SPL increases by 0.52%. Compared with the baseline, the introduction of the HEM module increases SR from 71.52% to 73.31%, indicating that this module plays an important role in capturing global dependency relationships and enhancing the semantics of historical scenes.

To verify the effectiveness of our proposed history modeling approach, we conduct a comprehensive ablation study on the R2R dataset. We compare our CA-VLN with several variants. The results are summarized in Table 7.

The ablation shows that Textual Retrieval Only performs the worst, as it lacks spatial structure and fine-grained visual grounding. This confirms that fusing hierarchical history allows the agent to make more informed decisions. By balancing global context and local details, our approach effectively prevents memory explosion while maintaining high navigation accuracy.

**Figure 6:**
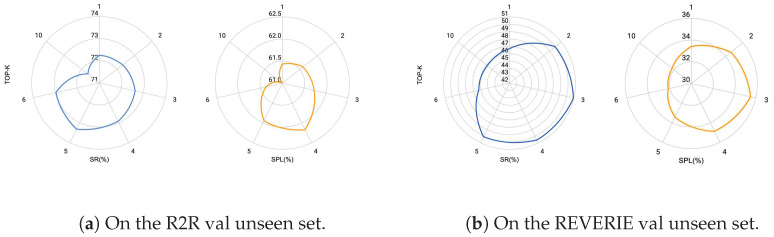
The Top-K hyperparameter analysis on the R2R and REVERIE Dataset.

As illustrated in Figure 6, the impact of the Top-K hyperparameter on model performance can be observed. When Top-K ranges from 3 to 5, both SR and SPL remain at excellent levels. However, when Top-K >5, SR of CA-VLN exhibits a marked downward trend. This can be linked to the introduction of redundant information as well as hallucination errors. In practice, we set Top-K =5 for the R2R and SOON datasets, and Top-K =3 for the REVERIE dataset. Additionally, the performance on the two datasets shows slight differences. Owing to the high-level semantic instructions inherent in the REVERIE dataset, model performance is more sensitive to redundant information, with more pronounced fluctuations in the evaluation metrics.

### 4.6. Qualitative Analysis

**Figure 7:**
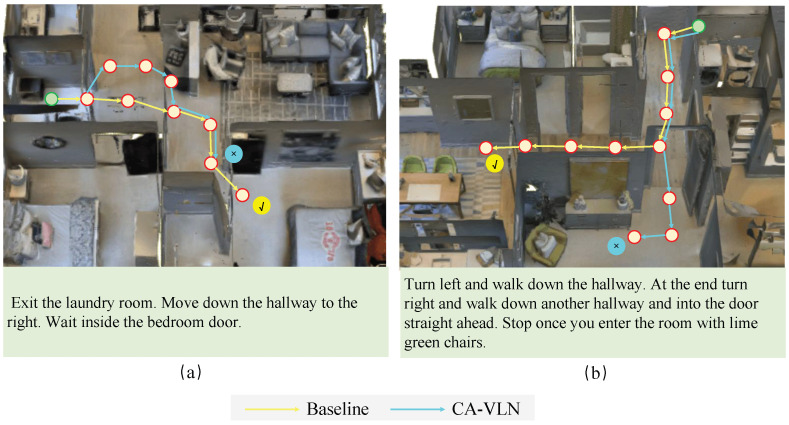
Qualitative comparison of CA-VLN and the baseline model on example images from the R2R dataset.

We present a qualitative comparison demonstrating the effectiveness of CA-VLN. Figure 7 illustrates a top-down trajectory diagram that exemplifies path planning dynamics. The path generated by CA-VLN demonstrates remarkable clarity and precision, faithfully following instructions to reach the target location. In Figure 7a, the instruction is accurately reflected in the planned path. In contrast, the baseline model exhibits navigational inaccuracies, presumably due to its inability to resolve the entity-specific semantic details of “hallway,” resulting in suboptimal path selection.

In Figure 7b, the CA-VLN agent successfully executes complex multi-step instructions such as “Turn left and walk down the hallway” and “walk down another hallway and into the door straight ahead,” ultimately arriving at the room containing “green chairs.” Through episodic memory enhancement, the agent leverages historical trajectory to avoid redundant movements. Simultaneously, the visual-semantic enhancement module enables task-relevant attention focusing (e.g., the green-chair room), thereby optimizing both planning and execution efficiency. The baseline model produces oversimplified and suboptimal trajectories that deviate from the intended path.

**Figure 8:**
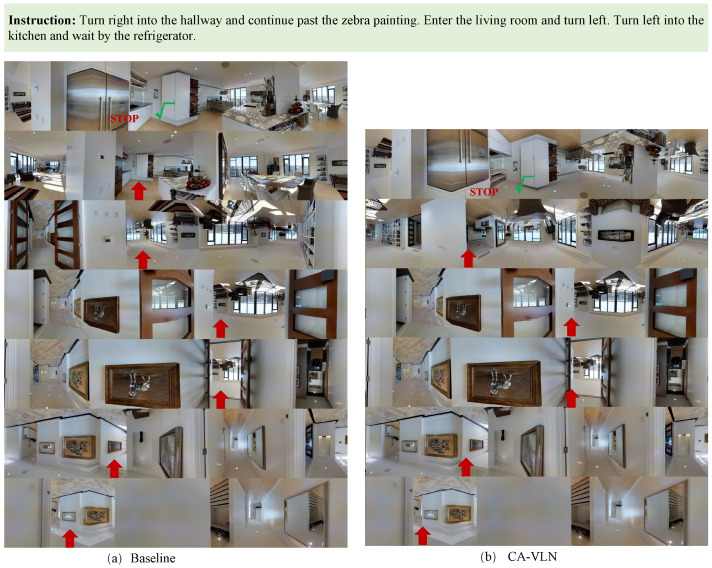
Example of the first-person perspective trajectory.

To evaluate the effectiveness, an example of the first-person perspective trajectory is given, as shown in Figure 8. The baseline model has more path turns when executing instructions because it lacks an understanding of details, while the CA-VLN is more efficient and has stronger adaptability through multimodal fusion and episodic memory optimization.

## 5. Conclusions and Discussion

We propose the CA-VLN framework, leveraging MLLMs to solve data scarcity, shallow multimodal fusion, and insufficient historical context issues in VLN tasks. Our dual-agent architecture includes a Knowledge Reasoning Agent enhancing instruction interpretation via knowledge integration, a Hierarchical History Agent enabling planning via episodic memory and a Knowledge-Guided Fusion Module to bridge MLLM-VLN gaps. Extensive experiments demonstrate the superiority of our approach. For example, on the R2R unseen test set, our CA-VLN achieves a SR of 73.31%, outperforming the recent state-of-the-art models KERM. This highlights the effectiveness of our MLLM-powered knowledge reasoning and hierarchical history management.

Our model excels in scenarios requiring complex, multi-step reasoning and backtracking, where the Hierarchical History Agent proves crucial. For instance, in environments with ambiguous hallways or recurring room layouts, our agent successfully navigated by referencing its episodic memory to avoid loops, a common failure point for previous models. However, the model’s performance is less optimal in visually sparse environments where few distinct landmarks are available. In such cases, the Knowledge Reasoning Agent struggles to extract meaningful semantic entities, leading to less effective instruction enhancement. This suggests that future work could focus on improving the agent’s intrinsic visual feature extraction capabilities to complement the knowledge-based approach, perhaps through self-supervised pre-training on a wider variety of visual data.

Finally, we address the challenge of deploying such models in physical environments, known as the Sim-to-Real gap. While our experiments are conducted in the Matterport3D simulator, the reliance on high-level semantic reasoning (via the Knowledge Agent) rather than low-level texture matching offers a promising pathway for real-world transferability. In physical settings, visual appearances vary drastically due to lighting and camera noise, but semantic concepts (e.g., the spatial relationship that “a sink is typically found in a kitchen”) remain invariant. By grounding navigation actions in these stable semantic concepts and maintaining a topological history, our CA-VLN framework is theoretically more robust to domain shifts than traditional end-to-end approaches. Future work will focus on validating this hypothesis by deploying the distilled Hierarchical History Agent on a mobile robot platform in unseen office environments.

## Figures and Tables

**Table 1:** Performance comparison between CA-VLN and existing discrete VLN methods on the R2R.

Moudels	Val_Seen				Val_Unseen				Test_Unseen			
	**TL**↓	**NE**↓	**SR**↑	**SPL**↑	**TL**↓	**NE**↓	**SR**↑	**SPL**↑	**TL**↓	**NE**↓	**SR**↑	**SPL**↑
Seq2seq [8]	11.33	6.01	39	-	8.39	7.81	22	-	8.13	7.85	20	18
RCM [58]	10.65	3.53	67	-	11.46	6.09	43	-	9.48	4.21	60.5	59
*VLN with Pretraining*												
RecBERT [21]	11.13	2.90	72	68	12.01	3.93	63	57	12.35	4.09	63	57
HAMT [19]	11.15	2.51	76	72	11.46	3.62	66	61	12.27	3.93	65	60
HOP+ [20]	11.31	2.33	78	73	11.76	3.49	67	61	12.67	3.71	66	60
ESceme [59]	10.65	2.57	76	73	10.80	3.39	68	64	11.89	3.77	66	63
KERM [48]	12.16	2.19	80	74	13.54	3.22	72	61	14.60	3.61	70	59
ACME [30]	11.28	2.16	80.12	**75.68**	12.23	3.12	72.75	**62.3**	13.43	3.68	**70.4**	**61.2**
VPNet [60]	-	3.37	66.69	62.13	-	5.43	51.23	43.47	-	5.11	52.40	42.87
*baseline:*												
DUET [22]	12.31	2.28	78.84	72.89	13.94	3.31	71.52	60.42	14.74	3.65	69.25	58.68
*VLN with LLMs*												
NaviLLM (Vicuna-7B) [36]	-	-	-	-	12.81	3.51	67	59	13.21	3.71	68	60
NavCot (LLaMA2-7B) [6]	10.08	6.46	41	38	9.95	6.26	40	37	-	-	-	-
CA-VLN	11.84	**2.04**	**81.03**	75.15	12.70	**3.03**	**73.31**	61.95	14.41	**3.37**	70.27	60.31

**Table 2:** Experimental results on the REVERIE dataset.

Moudels	Val_Unseen						Test_Unseen					
	**Navigation**				**Grounding**		**Navigation**				**Grounding**	
	TL ↓	OSR ↑	SR ↑	SPL ↑	RGSR ↑	RGSPL ↑	TL ↓	OSR ↑	SR ↑	SPL ↑	RGSR ↑	RGSPL ↑
Seq2seq [8]	11.07	8.07	4.20	2.84	2.16	1.63	10.89	6.88	3.99	3.09	2.00	1.58
RCM [58]	11.98	14.23	9.29	6.97	4.89	3.89	10.60	11.68	7.84	6.67	3.67	3.14
RecBERT [21]	16.78	35.02	30.67	24.90	18.77	15.27	15.86	32.91	29.61	23.99	16.50	13.51
HAMT [19]	14.08	36.84	32.95	30.20	18.92	17.28	13.62	33.41	30.40	26.67	14.88	13.08
DUET [22]	22.65	51.12	46.46	33.18	32.49	22.40	21.43	56.87	52.42	35.98	31.67	21.87
ACME [30]	23.38	53.97	49.46	32.37	32.64	24.02	22.77	57.48	51.89	34.65	**33.12**	23.57
KERM [48]	21.85	52.21	50.44	35.38	34.51	24.45	17.32	57.58	52.43	**39.21**	32.39	**23.64**
NaviLLM (Vicuna-7B) [36]	16.04	53.74	44.56	**36.63**	-	-	16.39	56.21	43.49	34.45	-	-
CA-VLN	23.47	**56.31**	**50.99**	35.54	**35.34**	**24.98**	21.52	**57.61**	**53.43**	37.28	32.86	23.59

**Table 3:** Experimental results on the SOON dataset.

Moudels	Val Unseen					Test Unseen				
	**TL**↓	**OSR**↑	**SR**↑	**SPL**↑	**RGSPL**↑	**TL**↓	**OSR**↑	**SR**↑	**SPL**↑	**RGSPL**↑
GBE [53]	28.96	28.54	19.52	13.34	1.16	27.88	21.45	12.90	9.23	0.45
NaviLLM (Vicuna-7B) [36]	28.66	33.11	19.81	14.29	-	-	-	-	-	-
DUET [22]	36.43	51.01	36.30	22.37	3.73	41.91	42.95	33.23	21.15	4.11
KERM [48]	35.83	51.62	38.05	23.16	4.04	-	-	-	-	-
ACME [30]	27.82	50.73	**38.43**	**27.81**	**4.33**	27.33	43.51	**35.55**	22.28	**5.47**
CA-VLN	39.67	**54.13**	37.32	21.90	3.60	39.95	**44.87**	34.35	**22.64**	5.26

**Table 4:** Ablation results of each module on the R2R validation set. CoT stands for data augmented through multimodal reasoning. Entity refers to Entity-Guided Feature Enhancement. Fusion represents the Knowledge-Guided Multimodal Fusion Module. History denotes history enhanced by situational memory retrospection.

Moudels	CoT	Entity	Fusion	History	Val_Seen				Val_Unseen			
					**TL**↓	**NE**↓	**SR**↑	**SPL**↑	**TL**↓	**NE**↓	**SR**↑	**SPL**↑
Baseline					12.31	2.28	78.84	72.89	13.94	3.31	71.52	60.42
1	✓				12.24	2.23	78.92	72.96	13.44	3.25	72.03	61.05
2		✓			12.16	2.24	78.97	73.01	13.56	3.19	72.18	61.17
3			✓		11.33	2.12	80.06	74.89	12.34	3.17	72.75	62.02
4				✓	12.17	2.09	80.61	75.01	12.71	3.21	72.35	61.58
5	✓	✓			12.03	2.21	79.13	73.32	12.82	3.09	72.26	61.45
6			✓	✓	12.22	2.05	80.70	74.46	12.50	3.07	72.97	62.11
7	✓	✓	✓	✓	12.05	2.20	80.21	74.63	12.83	3.15	72.80	61.43
8	✓	✓			12.58	2.11	80.42	74.30	12.75	3.18	72.56	61.23
9	✓	✓	✓	✓	11.84	**2.04**	**81.03**	**75.15**	12.70	**3.03**	**73.31**	61.95

**Table 5:** Ablation results of world knowledge fusion on the REVERIE validation set.

Moudels	KVSI	IGFF	Val_Unseen				
			**SR**↑	**OSR**↑	**SPL**↑	**RGSR**↑	**RGSPL**↑
Baseline			46.46	51.12	33.18	32.49	22.40
1	✓		49.76	54.58	34.96	34.11	23.57
2		✓	50.34	55.63	35.08	34.26	24.15
3	✓	✓	**50.99**	**56.31**	**35.54**	**35.34**	**24.98**

**Table 6:** Ablation results of Episodic Memory Enhancement Module on the R2R dataset.

Moudels	HHR	HEM	Val_Unseen			
			**TL**↓	**NE**↓	**SR**↑	**SPL**↑
Baseline			12.83	3.15	72.80	61.43
1	✓		12.94	3.11	73.05	61.67
2		✓	**12.68**	3.08	73.17	61.93
3	✓	✓	12.70	**3.03**	**73.31**	**61.95**

**Table 7:** Experiments of history modeling approaches on the R2R dataset.

	Val Seen				Val Unseen			
**Models**	**TL**↓	**NE**↓	**SR**↑	**SPL**↑	**TL**↓	**NE**↓	**SR**↑	**SPL**↑
topological history	12.31	2.28	78.84	72.89	13.94	3.31	71.52	60.42
simple text retrieval	10.56	4.62	68	62.37	9.37	4.51	64	59
hierarchical text retrieval	10.79	4.17	72.7	68.2	12.81	3.71	66	60
**Ours**	11.84	**2.04**	**81.03**	**75.15**	12.70	**3.03**	**73.31**	**61.95**

## Data Availability

The datasets used in this manuscript are publicly available datasets. Detailed information about these datasets is provided in Section 4.1 Dataset of this manuscript.

## Abbreviations

The following abbreviations are used in this manuscript:

CA-VLN	Collaborative Agents in Visual-Language Navigation
KVSI	Knowledge-aware Visual Semantic Interactor
IGFF	Instruction-Guided Feature Fusion
HHR	Hierarchical Historical Retrieval
HEM	History Enhancement Module

## Appendix A. Computational Complexity Analysis

While our dual-agent architecture introduces additional modules, the computational overhead remains within a feasible range for real-time applications.

Specific to our contributions:

- **Inference Latency:** The *History Agent* utilizes a lightweight Graph Attention Network (GAT). The graph size *N* is kept small, resulting in a negligible latency increase of ≈4 ms per step. The *Knowledge Agent* performs retrieval [50] via pre-computed embeddings, adding ≈12 ms. Total inference time per step increases by approximately 15% compared to the baseline, which is an acceptable trade-off for the significant gains in navigation success rate.
- **Memory Footprint:** The episodic memory graph stores compact feature vectors rather than raw images.
- **Efficiency:** Although the per-step cost is slightly higher, CA-VLN achieves a higher SPL (Success weighted by Path Length), indicating that the agent takes more direct paths to the goal. This reduces the *total* number of steps required for a trajectory, partially offsetting the per-step latency in long-horizon tasks.

## Appendix B. Algorithm of Multimodal Fusion and Action Decision

**Algorithm A1** Multimodal Fusion and Action Decision	
1:	**Input:** Observation $O_{t}$, Instruction *I*, Key Entities *E*, Knowledge *K*, Hierarchical History $H_{hist}$, Current Node Graph $G_{t}$
2:	**Output:** Action $a_{t}$
3:	**Parameter:**$\;T_{max}$ (Maximum steps)
4:	**Initialize:** t←0
5:	**while** ¬Agent.is_at_target() $\wedge t<T_{max}$**do**
6: *// 1. Feature Encoding*	
7: $V_{emb}\leftarrow \mathrm{ViT}\left(O_{t}\right)$	
8: $I_{emb},E_{emb},K_{emb}\leftarrow \mathrm{BERT}\left(I,E,K\right)$	
9: $h_{node}\leftarrow \mathrm{GAT}\left(G_{t}\right)$	
10: $h_{hist}\leftarrow \mathrm{BERT}\left(H_{hist}\right)$	
11: *// 2. Intermediate Representation & Fusion*	
12: **Module: HEM (History Encoding Module)**	
13: $x_{hem}\leftarrow \mathrm{Linear}\left(\mathrm{Concat}\left(h_{node},h_{hist}\right)\right)$	
14: ${\tilde{H}}_{t}\leftarrow 4*\mathrm{FFN}\left(x_{hem}\right)$	
15: **Module: Instruction-Entity Fusion**	
16: $ctx_{txt}\leftarrow \mathrm{MultiHeadAttn}\left(Q=I_{emb},K=E_{emb},V=E_{emb}\right)$	
17: $I_{t}^{\prime }\leftarrow \mathrm{Gate}\left(ctx_{txt},I_{emb}\right)$	
18: **Module: KVSI (Knowledge-Visual Semantic Interaction)**	
19: $x_{kvsi}\leftarrow \mathrm{CrossAttn}\left(Q=V_{emb},K=K_{emb},V=K_{emb}\right)$	
20: $x_{kvsi}\leftarrow \mathrm{SelfAttn}\left(x_{kvsi}\right)$	
21: $O_{t}^{\prime }\leftarrow 2*\mathrm{FFN}\left(x_{kvsi}\right)$	
22: *// 3. Global-Local Feature Fusion*	
23: **Module: IGFF (Instruction-Guided Feature Fusion)**	
24: $x_{igff}\leftarrow \mathrm{Linear}\left(O_{t}^{\prime }\right)$	
25: $x_{igff}\leftarrow \mathrm{CrossAttn}\left(Q=x_{igff},K=I_{t}^{\prime },V=I_{t}^{\prime }\right)$	
26: $F_{local}\leftarrow 2*\mathrm{FFN}\left(x_{igff}\right)$	
27: *// 4. Action Prediction*	
28: **Global Action Prediction:**	
29: $g_{t}\leftarrow \mathrm{CrossAttn}\left(Q={\tilde{H}}_{t},K=I_{t}^{\prime },V=I_{t}^{\prime }\right)$	
30: $Score_{global}\leftarrow 2*\mathrm{FFN}\left(\mathrm{GASA}\left(g_{t},G_{t}\right)\right)$	
31: **Local Action Prediction:**	
32: $l_{t}\leftarrow \mathrm{CrossAttn}\left(Q=F_{local},K=V_{emb},V=V_{emb}\right)$	
33: $Score_{local}\leftarrow 2*\mathrm{FFN}\left(\mathrm{SelfAttn}\left(l_{t}\right)\right)$	
34: *// 5. Decision Making*	
35: $Probs\leftarrow \mathrm{Softmax}(\left[Score_{global},Score_{local}\right])$	
36: $a_{t}\leftarrow \mathrm{ArgMax}\left(Probs\right)$	
37: **Execute** $a_{t}$; **Update**t←t+1, $H_{hist},G_{t}$	
30:	**end whilereturn** Execution Trajectory

## Appendix C. Replace ± with Std of 3 Runs

Performance comparison between CA-VLN and existing discrete VLN methods on the R2R dataset. We report the mean and standard deviation (±) over three independent training runs for our method to demonstrate stability (Table A1).

**Table A1:** Experiments on the R2R dataset.

	Val Seen				Val Unseen				Test Unseen			
**Models**	**TL**↓	**NE**↓	**SR**↑	**SPL**↑	**TL**↓	**NE**↓	**SR**↑	**SPL**↑	**TL**↓	**NE**↓	**SR**↑	**SPL**↑
Seq2Seq [8]	11.33	6.01	39	-	8.39	7.81	22	-	8.13	7.85	20	18
DUET [22]	12.31	2.28	78.84	72.89	13.94	3.31	71.52	60.42	14.74	3.65	69.25	58.68
NaviLLM [36]	-	-	-	-	12.81	3.51	67	59	13.21	3.71	68	60
**CA-VLN (Ours)**	11.84	**2.04**	**81.03**	**75.15**	12.70	**3.03**	**73.31**	**61.95**	14.41	**3.37**	**70.27**	**60.31**
*std (±)*	*0.15*	*0.05*	*0.08*	*0.21*	*0.19*	*0.08*	*0.12*	*0.15*	*0.17*	*0.09*	*0.08*	*0.21*

## References

[1] Huang W., Xia F., Xiao T., Chan H., Liang J., Florence P., Zeng A., Tompson J., Mordatch I., Chebotar Y. (2022). Inner Monologue: Embodied Reasoning through Planning with Language Models. arXiv.

[2] Driess D., Xia F., Sajjadi M.S.M., Lynch C., Chowdhery A., Ichter B., Wahid A., Tompson J., Vuong Q., Yu T. PaLM-E: An Embodied Multimodal Language Model. Proceedings of the International Conference on Machine Learning.

[3] OpenAI (2023). Gpt-4 Technical Report.

[4] Zhou G., Hong Y., Wu Q. (2024). Navgpt: Explicit reasoning in vision-and-language navigation with large language models. Proc. Aaai Conf. Artif. Intell..

[5] Zhou G., Hong Y., Wang Z., Wang X.E., Wu Q. Navgpt-2: Unleashing navigational reasoning capability for large vision-language models. Proceedings of the European Conference on Computer Vision (ECCV).

[6] Lin B., Nie Y., Wei Z., Chen J., Ma S., Han J., Xu H., Chang X., Liang X. (2025). NavCoT: Boosting LLM-Based Vision-and-Language Navigation via Learning Disentangled Reasoning. IEEE Trans. Pattern Anal. Mach. Intell..

[7] Zhao Q., Lu Y., Kim M.J., Fu Z., Zhang Z., Wu Y., Li Z., Ma Q., Han S., Finn C. CoT-VLA: Visual Chain-of-Thought Reasoning for Vision-Language-Action Models. Proceedings of the IEEE/CVF Conference on Computer Vision and Pattern Recognition (CVPR).

[8] Anderson P., Wu Q., Teney D., Bruce J., Johnson M., Sünderhauf N., Reid I., Gould S., van den Hengel A. Vision-and-Language Navigation: Interpreting Visually-Grounded Navigation Instructions in Real Environments. Proceedings of the 2018 IEEE/CVF Conference on Computer Vision and Pattern Recognition (CVPR).

[9] Chen H., Suhr A., Misra D., Snavely N., Artzi Y. TOUCHDOWN: Natural Language Navigation and Spatial Reasoning in Visual Street Environments. Proceedings of the 2019 IEEE/CVF Conference on Computer Vision and Pattern Recognition (CVPR).

[10] Ku A., Anderson P., Patel R., Ie E., Baldridge J. (2020). Room-Across-Room: Multilingual Vision-and-Language Navigation with Dense Spatiotemporal Grounding. Proceedings of the 2020 Conference on Empirical Methods in Natural Language Processing (EMNLP).

[11] Liu C., Zhu F., Chang X., Liang X., Ge Z., Shen Y.D. Vision-language navigation with random environmental mixup. Proceedings of the IEEE International Conference on Computer Vision (ICCV).

[12] Fu T.J., Wang X.E., Peterson M.F., Grafton S.T., Eckstein M.P., Wang W.Y. Counterfactual vision-and-language navigation via adversarial path sampling. Proceedings of the European Conference on Computer Vision, ECCV.

[13] Li J., Tan H., Bansal M. Envedit: Environment editing for vision-and-language navigation. Proceedings of the IEEE/CVF Conference on Computer Vision and Pattern Recognition.

[14] Ramrakhya R., Undersander E., Batra D., Das A. Habitat-web: Learning embodied object-search strategies from human demonstrations at scale. Proceedings of the IEEE/CVF Conference on Computer Vision and Pattern Recognition.

[15] Ramakrishnan S.K., Gokaslan A., Wijmans E., Maksymets O., Clegg A., Turner J.M., Undersander E., Galuba W., Westbury A., Chang A.X. Habitat-matterport 3d dataset (hm3d): 1000 large-scale 3d environments for embodied ai. Proceedings of the Thirty-fifth Conference on Neural Information Processing Systems Datasets and Benchmarks Track.

[16] Deitke M., VanderBilt E., Herrasti A., Weihs L., Salvador J., Ehsani K., Han W., Kolve E., Farhadi A., Kembhavi A. (2022). ProcTHOR: Large-scale embodied AI using procedural generation. Adv. Neural Inf. Process. Syst..

[17] Fried D., Hu R., Cirik V., Rohrbach A., Andreas J., Morency L.P., Berg-Kirkpatrick T., Saenko K., Klein D., Darrell T. (2018). Speaker-follower models for vision-and-language navigation. Adv. Neural Inf. Process. Syst..

[18] Gopinathan M., Abu-Khalaf J., Suter D., Masek M. (2024). StratXplore: Strategic Novelty-seeking and Instruction-aligned Exploration for Vision and Language Navigation. Proceedings of the IEEE International Conference on Robotics and Automation (ICRA).

[19] Chen S., Guhur P.L., Schmid C., Laptev I. (2021). History Aware multimodal Transformer for Vision-and-Language Navigation. Adv. Neural Inf. Process. Syst..

[20] Qiao Y., Qi Y., Hong Y., Yu Z., Wang P., Wu Q. (2023). Hop+: History-enhanced and order-aware pre-training for vision-and-language navigation. IEEE Trans. Pattern Anal. Mach. Intell..

[21] Hong Y., Wu Q., Qi Y., Rodriguez-Opazo C., Gould S. Vln bert: A recurrent vision-and-language bert for navigation. Proceedings of the IEEE/CVF Conference on Computer Vision and Pattern Recognition.

[22] Chen S., Guhur P.L., Tapaswi M., Schmid C., Laptev I. Think Global, Act Local: Dual-scale Graph Transformer for Vision-and-Language Navigation. Proceedings of the IEEE/CVF Conference on Computer Vision and Pattern Recognition.

[23] Wang H., Wang W., Liang W., Xiong C., Shen J. Structured scene memory for vision-language navigation. Proceedings of the IEEE/CVF Conference on Computer Vision and Pattern Recognition.

[24] Chaplot D.S., Salakhutdinov R., Gupta A., Gupta S. Neural topological slam for visual navigation. Proceedings of the IEEE/CVF Conference on Computer Vision and Pattern Recognition.

[25] An D., Qi Y., Li Y., Huang Y., Wang L., Tan T., Shao J. (2023). BEVBert: Topo-Metric Map Pre-training for Language-guided Navigation. arXiv.

[26] Chen J., Lin B., Xu R., Chai Z., Liang X., Wong K.Y. (2024). Mapgpt: Map-guided prompting with adaptive path planning for vision-and-language navigation. Proceedings of the 62nd Annual Meeting of the Association for Computational Linguistics (Volume 1:
Long Papers).

[27] Krantz J., Gokaslan A., Batra D., Lee S., Maksymets O. Waypoint models for instruction-guided navigation in continuous environments. Proceedings of the IEEE/CVF International Conference on Computer Vision.

[28] Wang X., Xiong W., Wang H., Wang W.Y. Look before you leap: Bridging model-free and model-based reinforcement learning for planned-ahead vision-and-language navigation. Proceedings of the European Conference on Computer Vision (ECCV).

[29] Koh J.Y., Lee H., Yang Y., Baldridge J., Anderson P. Pathdreamer: A world model for indoor navigation. Proceedings of the IEEE/CVF International Conference on Computer Vision.

[30] Wu J., Wu C., Shen X., Wang L. (2025). Adaptive cross-modal experts network with uncertainty-driven fusion for vision–language navigation. Knowl.-Based Syst..

[31] Shah D., Osiński B., Levine S. (2023). Lm-nav: Robotic navigation with large pre-trained models of language, vision, and action. Proceedings of the Conference on Robot Learning.

[32] Qiao Y., Qi Y., Yu Z., Liu J., Wu Q. March in chat: Interactive prompting for remote embodied referring expression. Proceedings of the IEEE/CVF International Conference on Computer Vision.

[33] Yokoyama N., Ha S., Batra D., Wang J., Bucher B. VLFM: Vision-Language Frontier Maps for Zero-Shot Semantic Navigation. Proceedings of the IEEE International Conference on Robotics and Automation.

[34] Schumann R., Zhu W., Feng W., Fu T.J., Riezler S., Wang W.Y. (2024). Velma: Verbalization embodiment of llm agents for vision and language navigation in street view. Proc. Aaai Conf. Artif. Intell..

[35] Long Y., Li X., Cai W., Dong H. (2024). Discuss before moving: Visual language navigation via multi-expert discussions. Proceedings of the IEEE International Conference on Robotics and Automation.

[36] Zheng D., Huang S., Zhao L., Zhong Y., Wang L. Towards learning a generalist model for embodied navigation. Proceedings of the IEEE/CVF Conference on Computer Vision and Pattern Recognition.

[37] Peng B., Li C., He P., Galley M., Gao J. (2023). Instruction tuning with gpt-4. arXiv.

[38] Mu Y., Zhang Q., Hu M., Wang W., Ding M., Jin J., Wang B., Dai J., Qiao Y., Luo P. (2023). EmbodiedGPT: Vision-Language Pre-Training via Embodied Chain of Thought. Adv. Neural Inf. Process. Syst..

[39] Zhang R., Han J., Zhou A., Hu X., Yan S., Lu P., Li H., Gao P., Qiao Y. (2023). Llama-adapter: Efficient fine-tuning of language models with zero-init attention. arXiv.

[40] Touvron H., Martin L., Stone K., Albert P., Almahairi A., Babaei Y., Bashlykov N., Batra S., Bhargava P., Bhosale S. (2023). Llama 2: Open foundation and fine-tuned chat models. arXiv.

[41] Speer R., Chin J., Havasi C. (2017). Conceptnet 5.5: An open multilingual graph of general knowledge. Proc. Aaai Conf. Artif. Intell..

[42] Auer S., Bizer C., Kobilarov G., Lehmann J., Cyganiak R., Ives Z. Dbpedia: A nucleus for a web of open data. Proceedings of the The Semantic Web.

[43] Meyer C.M., Gurevych I. (2012). Wiktionary: A new rival for expert-built lexicons? Exploring the possibilities of collaborative lexicography. Electronic Lexicography.

[44] Shen S., Li C., Hu X., Xie Y., Yang J., Zhang P., Rohrbach A., Gan Z., Wang L., Yuan L. K-lite: Learning transferable visual models with external knowledge. Proceedings of the Neural Information Processing Systems, NeurIPS.

[45] Zhou K., Zheng K., Pryor C., Shen Y., Jin H., Getoor L., Wang X.E. (2023). Esc: Exploration with soft commonsense constraints for zero-shot object navigation. Proceedings of the International Conference on Machine Learning.

[46] Wang H., Chen A.G.H., Li X., Wu M., Dong H. (2024). Find what you want: Learning demand-conditioned object attribute space for demand-driven navigation. Adv. Neural Inf. Process. Syst..

[47] Pan B., Panda R., Jin S.Y., Feris R., Oliva A., Isola P., Kim Y. (2023). Langnav: Language as a perceptual representation for navigation. arXiv.

[48] Li X., Wang Z., Yang J., Wang Y., Jiang S. Kerm: Knowledge enhanced reasoning for vision-and-language navigation. Proceedings of the IEEE/CVF Conference on Computer Vision and Pattern Recognition.

[49] Krishna R., Zhu Y., Groth O., Johnson J., Hata K., Kravitz J., Chen S., Kalantidis Y., Li L.J., Shamma D.A. (2017). Visual genome: Connecting language and vision using crowdsourced dense image annotations. Int. J. Comput. Vis..

[50] Zhang P., Li X., Hu X., Yang J., Zhang L., Wang L., Choi Y., Gao J. (2021). VinVL: Making Visual Representations Matter in Vision-Language Models. arXiv.

[51] Devlin J., Chang M.W., Lee K., Toutanova K. (2019). BERT: Pre-training of Deep Bidirectional Transformers for Language Understanding. Proceedings of the 2019 Conference of the North American Chapter of the Association for Computational Linguistics: Human Language Technologies, Volume 1 (Long and Short Papers).

[52] Qi Y., Wu Q., Anderson P., Wang X., Wang W.Y., Shen C., van den Hengel A. REVERIE: Remote Embodied Visual Referring Expression in Real Indoor Environments. Proceedings of the IEEE/CVF Conference on Computer Vision and Pattern Recognition.

[53] Zhu F., Liang X., Zhu Y., Chang X., Liang X. SOON: Scenario oriented object navigation with graph-based exploration. Proceedings of the IEEE/CVF Conference on Computer Vision and Pattern Recognition.

[54] Radford A., Kim J.W., Hallacy C., Ramesh A., Goh G., Agarwal S., Sastry G., Askell A., Mishkin P., Clark J. (2021). Learning transferable visual models from natural language supervision. Proceedings of the International Conference on Machine Learning.

[55] Liu H., Li C., Wu Q., Lee Y.J. Visual Instruction Tuning. Proceedings of the International Conference on Neural Information Processing Systems, NeurIPS.

[56] Hu E.J., Shen Y., Wallis P., Allen-Zhu Z., Li Y., Wang S., Wang L., Chen W. LoRA: Low-Rank Adaptation of Large Language Models. Proceedings of the International Conference on Learning Representations.

[57] Tan H., Bansal M. (2019). LXMERT: Learning Cross-Modality Encoder Representations from Transformers. Proceedings of the Conference on Empirical Methods in Natural Language Processing.

[58] Wang X., Huang Q., Celikyilmaz A., Gao J., Shen D., Wang Y.F., Wang W.Y., Zhang L. Reinforced cross-modal matching and self-supervised imitation learning for vision-language navigation. Proceedings of the IEEE Conference on Computer Vision and Pattern Recognition.

[59] Qi Z., Daqing L., Chaoyue W., Jing Z., Dadong W., Dacheng T. (2025). ESceme: Vision-and-Language Navigation with Episodic Scene Memory. Int. J. Comput Vis..

[60] Feng S., Wang Z., Li Y., Kong R., Cai H., Wang S., Lee G.H., Li P., Jiang S. VPN: Visual Prompt Navigation. Proceedings of the AAAI Conference on Artificial Intelligence.

